# A presentation of drowning detection system on coastal lines using image processing techniques and neural network 

**Published:** 2019-07

**Authors:** Kamyar Shiuuee, Fardin Rezaei

**Affiliations:** ^*a*^Applied Research Office, Guilan Commander of Law Enforcement Force, Rasht, Iran.; ^*b*^Deputy of Information Technology, Guilan Commander of Law Enforcement Force, Rasht, Iran.

## Abstract

**Background:**

Given the increased casualties of swimming at sea, there is an urgent need for an intelligent warning drowning detection system in the coasts. In order to provide such a system, image processing techniques in combination with the artificial intelligence are used. These challenges include the high noise of sea images, the presence of frequent moves in the image, and the size of the drowned from far distance.

**Methods:**

In this article, for monitoring the sea and automatic drowning detection, the swimmer is detected and the features of images are extracted through using image processing techniques and background omission. Then, using the artificial neural network, a network training and testing is done, and the drowning swimmer will be detected. This neural network of the perceptron type contains two hidden layers. There are 7 neurons in the first layer and 3 neurons in the second layer.

**Results:**

The results indicated that the neural network has detected drowning cases with the precision of 94.46%.

**Conclusions:**

For detecting drowning in sea beaches, the neural network is the best approach with the highest precision.

**Keywords:**

Drowning Detection, Coastal Warning, Image Processing, Neural Network

